# Plasma uric acid is related to large arterial stiffness but not to other hemodynamic variables: a study in 606 normotensive and never-medicated hypertensive subjects

**DOI:** 10.1186/s12872-021-02072-9

**Published:** 2021-05-26

**Authors:** Humam Hamid, Venla Kurra, Manoj Kumar Choudhary, Heidi Bouquin, Onni Niemelä, Mika A. P. Kähönen, Jukka T. Mustonen, Ilkka H. Pörsti, Jenni K. Koskela

**Affiliations:** 1 Faculty of Medicine and Health Technology, Tampere University, FI-33014 Tampere, Finland; 2 Laboratory and Medical Research Unit, Seinäjoki Central Hospital, Seinäjoki, Finland; 3 Department of Clinical Physiology, Tampere University Hospital, Tampere, Finland; 4 Department of Internal Medicine, Tampere University Hospital, Tampere, Finland

**Keywords:** Arterial stiffness, Hemodynamics, Impedance cardiography, Pulse wave analysis, Uric acid

## Abstract

**Background:**

Elevated level of plasma uric acid (PUA) has been associated with cardiovascular disease, but whether uric acid is an independent risk factor or merely a marker remains controversial.

**Methods:**

We investigated in a cross-sectional setting the association of PUA with hemodynamics in 606 normotensive and never-medicated hypertensive subjects (295 men, 311 women, age range 19–73 years) without cardiovascular disease or gout. In all except 15 individuals, PUA was within the normal range. Supine hemodynamics were recorded using whole-body impedance cardiography and radial tonometric pulse wave analysis.

**Results:**

The mean concentrations of PUA in age, sex and body mass index adjusted quartiles were 234, 278, 314, and 373 µmol/l, respectively. The highest PUA quartile presented with higher aortic to popliteal pulse wave velocity (PWV) than the lowest quartile (8.7 vs. 8.2 m/s, *p* = 0.026) in analyses additionally adjusted for plasma concentrations of C-reactive protein, low density lipoprotein cholesterol, triglycerides, and mean aortic blood pressure. No differences in radial and aortic blood pressure, wave reflections, heart rate, cardiac output, and systemic vascular resistance were observed between the quartiles. In linear regression analysis, PUA was an independent explanatory factor for PWV (β = 0.168, *p* < 0.001, R^2^ of the model 0.591), but not for systolic or diastolic blood pressure. When the regression analysis was performed separately for men and women, PUA was an independent predictor of PWV in both sexes.

**Conclusions:**

PUA concentration was independently and directly associated with large arterial stiffness in individuals without cardiovascular disease and PUA levels predominantly within the normal range. *Trial registration* ClinicalTrials.gov NCT01742702.

## Background

Cardiovascular disease (CVD) is the leading cause of death worldwide [1]. The World Health Organization estimates that 17.9 million people died from CVDs in 2016, which represents 31% of all global deaths [1]. The role of uric acid, the final product of purine degradation [2], in the development and progression of CVDs has been a subject for an ongoing debate [3, 4].

An umbrella review analyzing systematic reviews, meta-analyses, and Mendelian randomization studies was recently conducted to investigate uric acid’s role in various health outcomes [3]. The outcome was that convincing evidence of a clear role for uric acid existed only regarding nephrolithiasis and gout, whilst suggestive evidence was observed with heart failure, hypertension, impaired fasting glucose or diabetes, chronic kidney disease, and coronary heart disease [3].

One possible link between uric acid and CVD is through arterial stiffness. Decreased large arterial compliance is an acknowledged prognostic marker for CVDs [5, 6]. The recording of pulse wave velocity (PWV) is regarded as the gold standard in the evaluation of large arterial stiffness [7]. In 1225 never-treated hypertensive patients plasma uric acid (PUA) levels were directly and independently associated with carotid-femoral PWV, but negatively associated with augmentation index (AIx), a variable of wave reflections [8]. In the Baltimore Longitudinal Study of Aging, higher serum uric acid was associated with greater increase in PWV in men but not in women during 6 years of follow-up [9]. However, this association was lost when men with PUA ≥ 370 µmol/l were excluded, suggesting a threshold for uric acid association with arterial stiffness [9]. A report from the Framingham heart study suggested that every 60 µmol/l increase in uric acid resulted in a 0.04 m/s (*p* = 0.016) increase in carotid-to-femoral PWV when hypertensive subjects were excluded [10]. A recent cross-sectional study found that PWV was higher in medicated hypertensive subjects with hyperuricemia than in hypertensive subjects without hyperuricemia [11]. Also a meta-analysis containing information from 24 publications supported the view that serum uric acid is related to higher carotid-femoral PWV in the general population [4]. However, the matter remains controversial, as serum uric acid was not, but uric acid-to-creatinine ratio in the urine was related to the risk of having higher PWV in 2296 Chinese subjects with a mean age of 43 years [12].

Previously the plasma concentration of uric acid has been related with the level of blood pressure [13], arterial stiffness [4, 9, 11, 14], wave reflections [8, 15], and markers of endothelial injury [16]. In this cross-sectional study our objective was to examine the association of uric acid with several functional hemodynamic variables in a generally healthy population with uric acid concentrations predominantly within the normal range. The present results suggest an independent relation between uric acid and large arterial stiffness, but no association between uric acid and blood pressure (BP), cardiac output, systemic vascular resistance, or wave reflection.

## Methods

### Study subjects

The recruitment of the study subjects has been previously described [17–19]. All subjects were examined by a physician, office BP was measured, and laboratory analyses were taken as previously described [20]. Laboratory samples were taken 12 (10, 13) days, and medical examination performed 8 (4, 13) days [mean (95% confidence internal, CI)] before the hemodynamic measurements. Medical history, lifestyle behavior and family history were documented. Alcohol use was evaluated as consumption of standard drinks (~ 12 g of absolute alcohol) per week and categorized to low, moderate, high according to the prevailing Finnish guidelines [21]. Smoking amount was estimated in pack-years. All subjects with BP lowering or uric acid level altering medications were excluded. The other exclusion criteria were history of coronary artery disease, stroke, cardiac insufficiency, valvular heart disease, chronic kidney disease, secondary hypertension, alcohol or substance abuse, psychiatric illness other than mild to moderate depression or anxiety, heart rhythm other than sinus rhythm, and ongoing pregnancy. Altogether 606 subjects, aged 20–72 years, participated in the study. The study was registered in two international databases of clinical studies (Eudra-CT registration number 2006-002065-39, ClinicalTrials.gov NCT01742702).

The following stable medications were used by the participants: female hormones for contraception or hormone replacement therapy (n = 63), antidepressants (40), vitamin D supplements (39), hormone-releasing intrauterine devices (24), thyroxin (21), inhaled glucocorticoids (17), antihistamines (16), statins (14), proton pump inhibitors (13), calcium supplementation (10), antirheumatics (6), low dose acetylsalicylic acid (6), anxiolytics (6), inhaled β_2_-mimetics (4), non-steroidal anti-inflammatory drugs (3), coxibs (3), pregabalin or gabapentin (3), antiepileptics (2), varenicline (2), warfarin (2), isotretinoin (1), ezetimibe (1), and tramadol (1).

### Laboratory analyses

Blood and urine samples were taken after about 12 h of fasting. Plasma C-reactive protein (CRP), sodium, potassium, glucose, creatinine, uric acid, triglyceride, and total, high-density (HDL) and low-density lipoprotein (LDL) cholesterol concentrations were determined using Cobas Integra 800 (F. Hoffmann-LaRoche Ltd, Basel, Switzerland). Insulin and parathyroid hormone (PTH) were determined using electrochemiluminescence immunoassay (Cobas e411, Roche Diagnostics). Estimated glomerular filtration rate (eGFR) was calculated using the CKD-EPI cystatin C equation [22], and insulin sensitivity was evaluated by the quantitative insulin sensitivity check index (QUICKI) [23]. Plasma renin activity (GammaCoat® Plasma Renin Activity 125-I RIA Kit, DiaSorin, Saluggia, Italy) and aldosterone concentration (Active® Aldosterone RIA, Beckman Coulter, Fullerton, CA, USA) were determined using commercial kits. Urine albumin excretion was determined using immunoturbidimetry.

The normal reference range for uric acid in Finland is 230–480 µmol/l for men, 155–350 µmol/l for women aged 18–49 years, and 155–400 µmol/l for women aged ≥ 50 years [24].

### Experimental protocol

Hemodynamic recordings were performed in a noiseless, temperature-controlled laboratory [17, 18, 25]. Products containing caffeine, smoking or heavy meal were to be avoided for ≥ 4 h, and alcohol consumption for ≥ 24 h before the investigation. The subjects rested supine on the examination table with impedance cardiography electrodes placed on body surface, tonometric sensor for pulse wave analysis on left radial pulsation, and oscillometric brachial cuff for BP calibration to the right upper arm. The left arm with the tonometric sensor was abducted to 90° in a support, which held the measurement probe at the heart level.

The measurement consisted of one 5-min period with continuous capture of hemodynamic data. For the analyses, the mean values of each 1-min period of recording were calculated. The repeatability and reproducibility of the protocol has been demonstrated to be good [17, 18, 25].

### Pulse wave analysis

Radial BP and pulse wave were continuously recorded by a tonometric sensor (Colin BP-508 T, Colin Medical Instruments Corp., USA) [17, 18]. The radial BP was calibrated approximately every 2.5 min by brachial BP measurements. Aortic BP was derived with the validated SphygmoCor pulse wave monitoring system (SpygmoCor PWMx, AtCor medical, Australia) [26]. Aortic pulse pressure and augmentation index (augmented pressure/pulse pressure * 100) were also determined. Central forward wave amplitude was defined as the difference between waveform foot and first systolic inflection point pressure in the aortic waveform [27, 28].

### Whole-body impedance cardiography

We used whole-body impedance cardiography (CircMon^R^, JR Medical Ltd., Tallinn, Estonia) that records changes in body electrical impedance during cardiac phases to measure heart rate, stroke volume, cardiac output, and PWV [29, 30]. Systemic vascular resistance was calculated from radial BP and cardiac output measured by CircMon^R^. Stroke volume, cardiac output and systemic vascular resistance were presented as indexes related to body surface area calculated using the DuBois equation [31]. The method and electrode configuration have been previously reported [30, 32]. Briefly, current electrodes were placed on the distal parts of the extremities proximally to the wrists and the ankles. Voltage electrodes were placed about 5 cm proximally to the current electrodes. To record the distal impedance from the popliteal artery at knee joint level, an active electrode was placed on the lateral side of the knee and the reference electrode on the calf about 20 cm apart. When the pressure wave enters the aortic arch and the diameter of the aorta changes, the voltage electrodes on the distal parts of the extremities record the decrease in whole-body impedance. PWV is calculated from the time difference between the onset of the decrease (‘foot’) in the whole-body impedance and the popliteal artery signal, and the distance between the electrodes [30, 32].

With CircMon^R^ the recorded stroke volume and cardiac output are in good agreement with values obtained utilizing 3 dimensional echocardiography [25] and the thermodilution and direct oxygen Fick methods [29, 32], and the PWV values show very good correlation with values measured using ultrasound or the tonometric method [30, 33].

### Statistics

The normally distributed data was analyzed using analysis of variance, non-normally distributed data using Kruskal-Wallis test with Mann–Whitney U-test in the post-hoc analyses. The Bonferroni correction was applied in all post-hoc analyses. IBM SPSS Statistics Version 26 (IBM Corporation, Armonk, NY, USA) was used for statistics. The results in the tables were presented as means and standard errors of the mean (SEM), or median [25th–75th percentiles], and in the figures as means and 95% confidence intervals of the mean. *p* < 0.05 was considered significant. The hemodynamic values were calculated as averages from the minutes 3–5 of the recordings when the signal was most stable. For the analyses, the subjects were divided into quartiles of PUA concentration that were adjusted for sex; or age, sex, and body mass index (BMI), as appropriate. Proportions on subjects in the PUA quartiles were compared using the Chi-square test. The use of the medications listed above did not differ between the quartiles of PUA.

For the statistical analyses, the continuous variables not normally distributed were Lg_10_-transformed. Linear regression analyses with stepwise elimination were used to investigate factors independently associated with hemodynamic variables. The covariates in the analyses were age, sex, BMI, alcohol consumption category (low, moderate, high) [21], smoking status (never, current, previous); plasma calcium, phosphate, PTH, Lg_10_ of triglycerides, HDL cholesterol, LDL cholesterol, uric acid, renin, aldosterone, Lg_10_ of CRP, sodium; Lg_10_ of QUICKI, eGFR, Lg_10_ of PWV, and mean aortic pressure, as appropriate.

## Results

### Study population and laboratory values

The PUA concentrations (mean ± SEM) were 344 ± 4 µmol/l in men and 258 ± 3 µmol/l in women (*p* < 0.001). The sex-adjusted demographic characteristics of the study participants according to PUA quartiles are shown in Table 1. Subject age was higher in quartile (Q) 4 versus Q1, while weight was higher in Q3 and Q4 than in Q1 and Q2. No difference was observed in height, whereas BMI was higher in Q3 than in Q2 and Q1 and highest of all in Q4.

**Table 1:** Results of the study participants in sex adjusted quartiles of fasting plasma uric acid concentrations

	Q1 n = 141	Q2 n = 155	Q3 n = 165	Q4 n = 144
Male/female (n)	74/77	73/79	76/77	72/78
Age (years)	43.7 (0.96)	44.3 (0.95)	44.3 (0.95)	47.5 (0.96)*
Weight (kg)	74.9 (1.2)	77.5 (1.2)	82.1 (1.2)*^†^	86.5 (1.2)*^†^
Height (cm)	173.0 (0.8)	172.9 (0.8)	173.2 (0.8)	172.8 (0.8)
Body mass index (kg/m^2^)	24.9 (0.33)	25.7 (0.33)	27.3 (0.32)*^†^	28.9 (0.33)*^†‡^

Results shown as mean (standard error of mean)

*p < 0.05 versus Q1; ^†^*p <* 0.05 versus Q2; ^‡^*p <* 0.05 versus Q3

Because of the above differences, the laboratory values in Table 2 are presented in sex, age and BMI adjusted quartiles of PUA, the concentrations of which were 234, 278, 314, and 373 µmol/l, respectively. Office systolic BP was higher in Q4 than in Q1, while office diastolic BP was higher in Q3 and Q4 than in Q1. The proportion of subjects with office hypertension (BP ≥ 140/90 mmHg) [20] was higher in Q3 than in Q1. No differences were observed in office heart rate, percentage of smokers, average alcohol intake, plasma electrolyte concentrations, or urine albumin excretion between the quartiles (Table 2). Plasma CRP was slightly higher in Q4 versus Q1 and Q2, while plasma renin activity, and plasma concentrations of aldosterone, PTH, and eGFR were corresponding in all quartiles. No differences were observed in fasting plasma total cholesterol, HDL cholesterol, glucose, and insulin, while QUICKI was lower in Q4 than in Q1. Plasma triglyceride concentration was higher in Q4 and Q3 when compared with Q1, and in Q4 versus Q2, whilst LDL cholesterol was somewhat higher in Q3 than in Q1.

**Table 2:** Clinical characteristics and laboratory results of the study participants in age, sex and body mass index adjusted quartiles of fasting plasma uric acid concentrations

	Q1 n = 141	Q2 n = 155	Q3 n = 165	Q4 n = 144
Office measurements				
Systolic BP (mmHg)	136 (1.5)	140 (1.5)	142 (1.5)	143 (1.5)*
Diastolic BP (mmHg)	87 (0.9)	89 (0.9)	90 (0.9)*	91 (0.9)*
Heart rate (bpm)	66.5 (0.8)	67.2 (0.8)	67.7 (0.8)	68.3 (0.9)
Number (%) of participants with BP ≥ 140/90 mmHg	68 (48.2)	92 (59.4)	106 (64.2)*	89 (61.8)
Current smokers (number)	20	18	18	19
Alcohol (standard drinks/week)	2 [0–6]	2 [0–5]	3 [1–5]	3 [1–7]
Uric acid (µmol/l)	234 (4)	278 (4)*	314 (4)*^†^	373 (4)*^†‡^
Sodium (mmol/l)	140 (0.2)	141 (0.2)	140 (0.2)	140 (0.2)
Potassium (mmol/l)	3.8 (0.0)	3.8 (0.0)	3.8 (0.0)	3.8 (0.0)
Calcium (mmol/l)	2.29 (0.01)	2.30 (0.01)	2.31 (0.01)	2.32 (0.01)
Phosphate (mmol/l)	0.95 (0.01)	0.97 (0.01)	0.96 (0.01)	0.99 (0.01)
C-reactive protein (mg/l)	0.5 [0.5–1.4]	0.8 [0.5–1.8]	1.0 [0.5–2.1]	1.0 [0.5-2.1]*^†^
Renin activity (ng Ang I/ml/h)	0.6 [0.3–1.1]	0.7 [0.4–1.2]	0.7 [0.5–1.3]	0.8 [0.5-1.3]
Aldosterone (pmol/l)	422 [292–569]	449 [329–609]	423 [320–572]	461 [338-620]
PTH (pmol/l)	4.32 (0.13)	4.51 (0.13)	4.75 (0.13)	4.66 (0.14)
eGFR (ml/min/1.73 m^2^)	102 (1.2)	100 (1.2)	97 (1.2)	98 (1.2)
Albumin excretion (µg/min)^a^	4 [3–5]	4 [3–6]	4 [3–5]	4 [3–5]
Total cholesterol (mmol/l)	5.0 (0.1)	5.0 (0.1)	5.2 (0.1)	5.2 (0.1)
Triglycerides (mmol/l)	0.9 [0.6–1.2]	1.0 [0.7–1.3]	1.1 [0.8–1.5]*	1.2 [0.8–1.8]*^†^
HDL cholesterol (mmol/l)	1.65 (0.03)	1.56 (0.03)	1.55 (0.03)	1.57 (0.03)
LDL cholesterol (mmol/l)	2.9 (0.1)	3.0 (0.1)	3.2 (0.1)*	3.1 (0.1)
Glucose (mmol/l)	5.4 (0.1)	5.4 (0.1)	5.4 (0.1)	5.5 (0.1)
Insulin (mU/l)	7.2 (1.4)	7.9 (1.4)	10.7 (1.4)	9.8 (1.5)
QUICKI	0.361 [0.342–0.381]	0.359 [0.367–0.376]	0.352 [0.332–0.372]	0.345 [0.324–0.373]*

Results shown as mean (standard error of mean) or median [27th–75th percentile]; *PTH* parathyroid hormone, *eGFR* cystatin C-based estimated glomerular filtration rate (CKD-EPI) [22], *HDL* high density lipoprotein, *LDL* low density lipoprotein, *QUICKI* quantitative insulin sensitivity check index

*p < 0.05 versus Q1; ^†^*p <* 0.05 versus Q2; ^‡^*p <* 0.05 versus Q3

^a^Albumin excretion results were available 114–130 subjects in each quartile

### Hemodynamic measurements

**Fig. 1:**
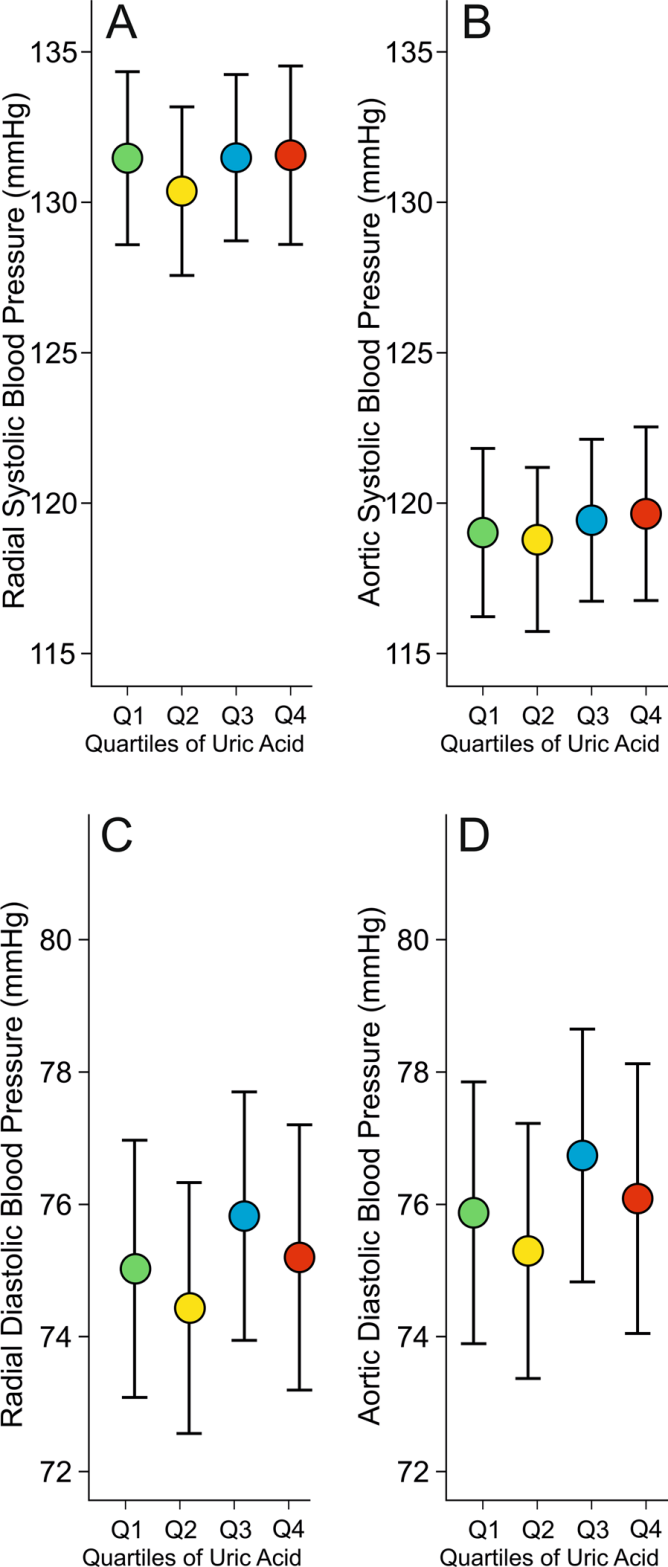
Radial (**A**) and aortic (**B**) systolic blood pressure, and radial (**C**) and aortic (**D**) diastolic blood pressure in 606 subjects presented in age, body mass index, sex, and plasma C-reactive protein, triglyceride, and LDL cholesterol adjusted quartiles of plasma uric acid concentration; mean ± 95% confidence interval of the mean

**Fig. 2:**
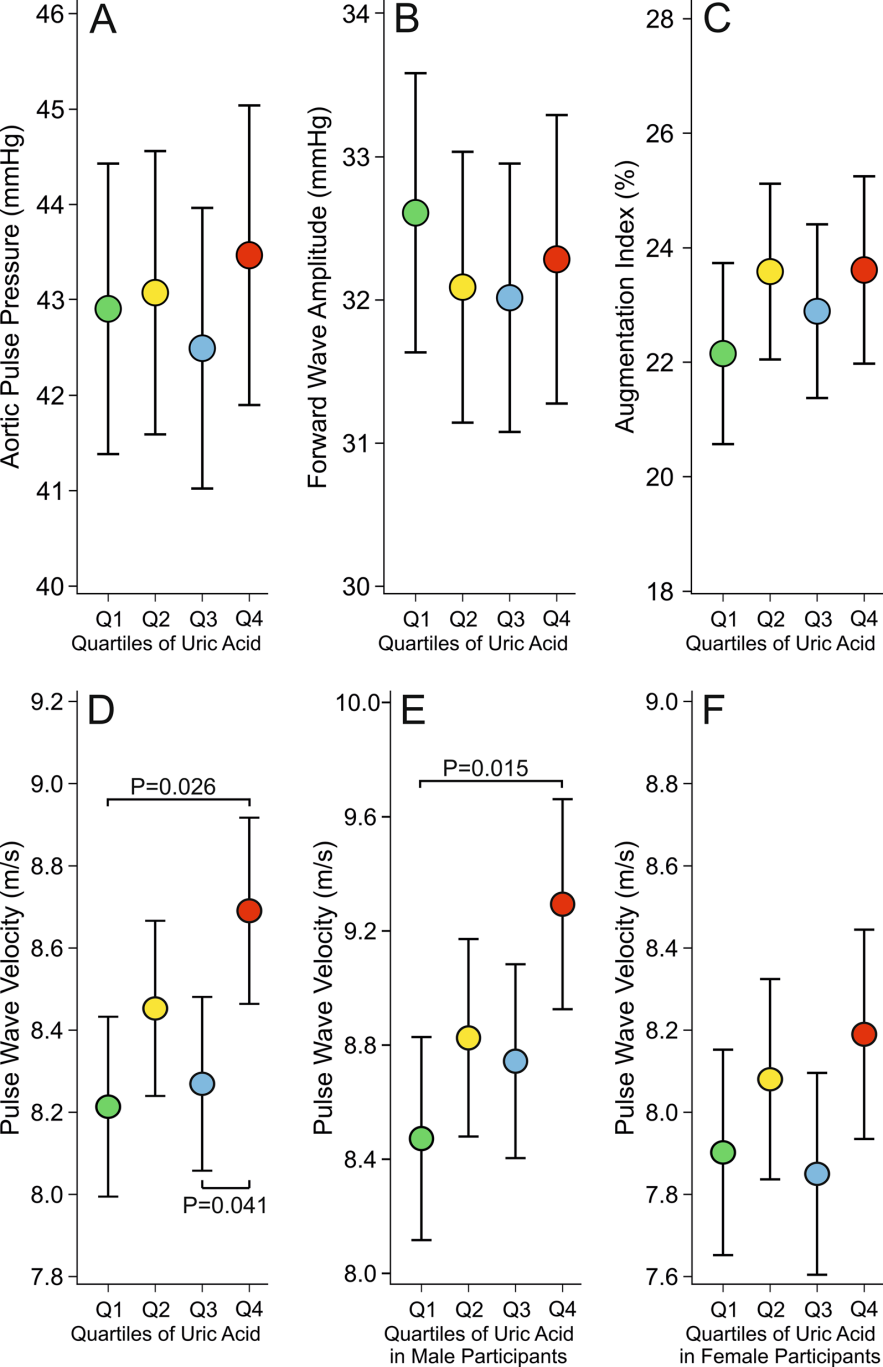
Aortic pulse pressure (**A**), forward wave amplitude (**B**), augmentation index (**C**), and pulse wave velocity (**D**) in 606 all subjects, and pulse wave velocity separately in men (**E**) and women (**F**), presented in age, body mass index, sex, C-reactive protein, triglyceride, and LDL cholesterol adjusted quartiles of plasma uric acid concentration; mean ± 95% confidence interval of the mean

**Fig. 3:**
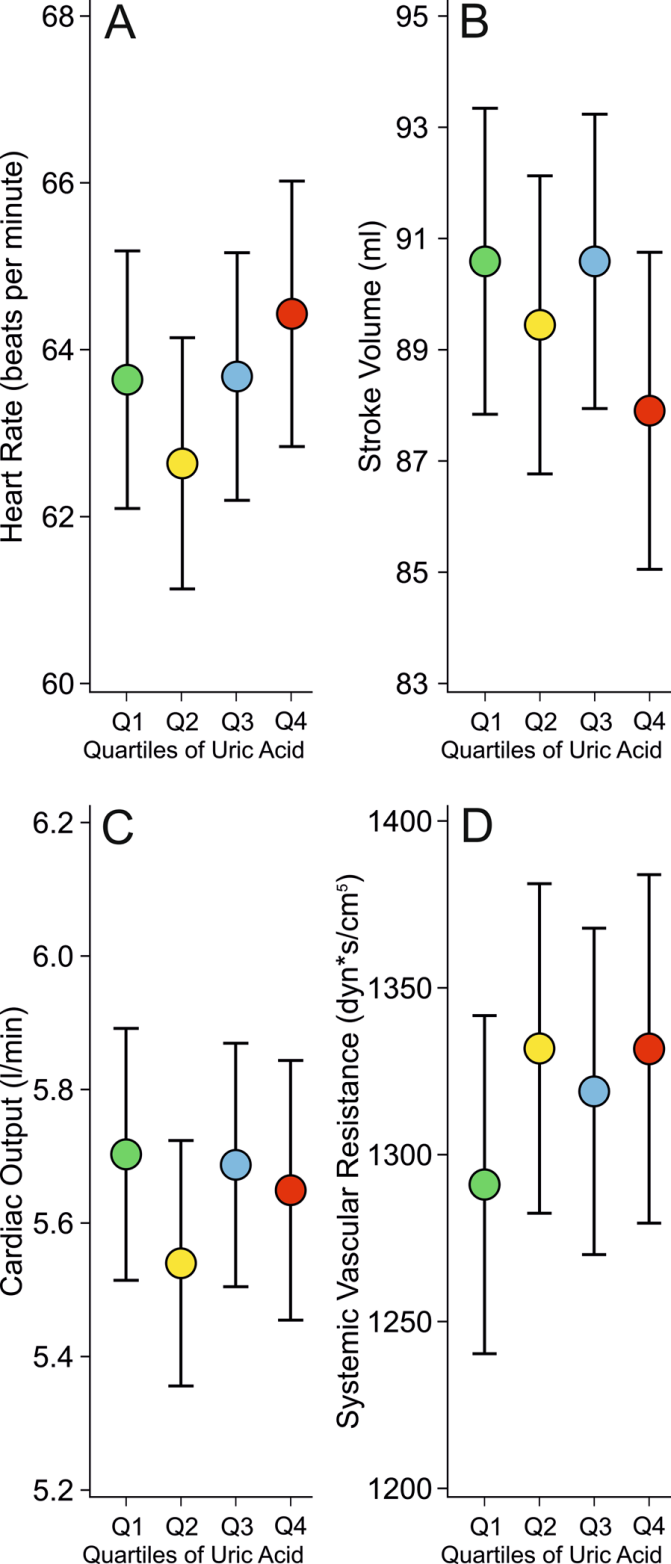
Heart rate (**A**), stroke volume (**B**), cardiac output (**C**), systemic vascular resistance (**D**) in 606 subjects presented in age, body mass index, sex, C-reactive protein, triglyceride, and LDL cholesterol adjusted quartiles of plasma uric acid concentration; mean ± 95% confidence interval of the mean

The results representing the hemodynamic variables in the age, sex and BMI adjusted quartiles of PUA are shown in Figs. 1, 2 and 3. No differences were observed in radial BP, calibrated from contralateral brachial BP measurements, or aortic BP between the quartiles (Fig. 1A–D).

Aortic pulse pressure, forward wave amplitude and augmentation index were similar in all quartiles (Fig. 2A–C), but aortic to popliteal PWV was higher in Q4 versus Q3 and Q1 (Fig. 2D). When analyzed separately in men and women, PWV was higher in Q4 versus Q1 in men (Fig. 2E), while in women no significant differences in PWV between the PUA quartiles were detected (Fig. 2F). The Pearson correlation between PUA concentration and PWV was 0.351 among all study subjects (*p* < 0.001), 0.338 in women (*p* < 0.001), and 0.242 in men (*p* < 0.001).

No differences were found between the quartiles in heart rate, stroke volume, cardiac output, and systemic vascular resistance (Fig. 3A–D).

### Hemodynamic variables in linear regression analyses

The results of the linear regression analyses with stepwise elimination are presented in Tables 3 and 4. The independent explanatory factors for aortic systolic (R^2^ = 0.399) and diastolic BP (R^2^ = 0.350) were PWV, eGFR, plasma concentrations of calcium and PTH, and QUICKI (Table 3). In addition, LDL cholesterol and triglycerides were independently associated with systolic BP, while male sex and high alcohol consumption category showed independent associations with diastolic BP (Table 3).

**Table 3:** Significant explanatory variables for aortic systolic and diastolic blood pressure in linear regression analysis with stepwise elimination

Systolic blood pressure (mmHg)	B	Beta	*p*	Diastolic blood pressure (mmHg)	B	Beta	*p*
R^2^ = 0.399				R^2^ = 0.350			
(constant)	− 24.905			(constant)	− 23.172		
Lg_10_ of pulse wave velocity	81.312	0.384	< 0.001	Lg_10_ of pulse wave velocity	47.287	0.333	< 0.001
eGFR	− 0.188	− 0.174	< 0.001	Calcium	20.013	0.159	< 0.001
Calcium	23.794	0.127	< 0.001	PTH	1.013	0.128	0.001
PTH	1.248	0.106	0.003	eGFR	− 0.115	− 0.159	< 0.001
LDL	3.173	0.150	< 0.001	Lg_10_ of QUICKI	− 34.464	− 0.127	0.001
Lg_10_ of QUICKI	− 40.982	− 0.102	0.007	High alcohol consumption category	7.565	0.083	0.020
Lg_10_ of triglycerides	− 7.905	− 0.091	0.030	Sex (male)	2.103	0.080	0.029

Variables in Model: Age, sex, body mass index, alcohol consumption category (low, moderate, high), smoking status (current, previous); plasma uric acid, sodium, calcium, phosphate, PTH, HDL cholesterol, LDL cholesterol, eGFR; Lg_10_ of triglycerides, C-reactive protein, renin, aldosterone, QUICKI, pulse wave velocity. *LDL* low-density lipoprotein, *HDL* high-density lipoprotein, *QUICKI* quantitative insulin sensitivity check index, *PTH* parathyroid hormone, *eGFR* estimated glomerular filtration rate from plasma cystatin-C using the CKD-EPI formula [15]

**Table 4:** Significant explanatory variables for aortic to popliteal pulse wave velocity in linear regression analysis with stepwise elimination

Pulse wave velocity (m/s)	B	Beta	*p*
Men and women, R^2^ = 0.591			
(constant)	7.594		< 0.001
Age	0.076	0.501	< 0.001
Ejection duration	− 0.020	− 0.223	< 0.001
Mean aortic pressure	0.022	0.180	< 0.001
Uric acid	0.004	0.168	< 0.001
Body mass index	0.032	0.077	0.019
Lg_10_ of triglycerides	0.557	0.069	0.034
Current smoking	− 0.363	− 0.067	0.015
Women, R^2^ = 0.580			
(constant)	− 0.025		
Age	0.073	0.524	< 0.001
Mean aortic pressure	0.023	0.221	< 0.001
Heart rate	0.032	0.187	< 0.001
Lg_10_ of triglycerides	1.309	0.173	< 0.001
Uric acid	0.003	0.090	0.032
Men, R^2^ = 0.532			
(constant)	7.747		< 0.001
Age	0.084	0.481	< 0.001
Ejection duration	− 0.024	− 0.230	< 0.001
Uric acid	0.006	0.171	< 0.001
Mean aortic pressure	0.025	0.164	0.001
LDL cholesterol	0.255	0.113	0.014

Variables in Model: Age, sex, body mass index, alcohol consumption category (low, moderate, high), smoking status (current, previous), mean aortic pressure, heart rate, ejection duration, eGFR, uric acid, HDL cholesterol, LDL cholesterol; Lg_10_ of triglycerides; Lg_10_ of C-reactive protein, and QUICKI. *HDL* high-density lipoprotein, *LDL* low-density lipoprotein, *QUICKI* quantitative insulin sensitivity check index, *eGFR* estimated glomerular filtration rate from plasma cystatin-C using the CKD-EPI formula [22]

The independent explanatory factors for PWV were age, ejection duration, mean aortic pressure, PUA, plasma triglycerides, BMI, and current smoking (R^2^ = 0.591) (Table 1). If subjects with PUA ≥ 370 µmol/l were excluded from the regression analysis [9], PUA concentration was still associated with PWV in the remaining 507 participants (beta = 0.003, *p* = 0.006). Altogether the present analyses suggested that for every 100 µmol/l increase in PUA, the associated increase in PWV was 0.9 m/s.

The explanatory variables for PWV were then analyzed separately for sexes (Table 4). In women, the explanatory variables for PWV were age, mean aortic pressure, heart rate, plasma triglycerides, and PUA (R^2^ = 0.580). In men, the explanatory variables for PWV were age, ejection duration, PUA, mean aortic pressure, BMI, and LDL cholesterol (R^2^ = 0.532).

In additional regression analyses, PUA was not an explanatory factor for forward wave amplitude, augmentation index, systemic vascular resistance, stroke volume, heart rate, or cardiac output (data not shown).

## Discussion

In this study, we investigated the association of uric acid with several cardiovascular variables in normotensive subjects and in never-medicated hypertensive patients without cardiovascular disease or gout. In analyses adjusted for confounding factors, PUA was significantly associated with PWV but not with any other hemodynamic variables including radial and aortic BP. The linear regression analyses confirmed that PUA was an independent explanatory factor for PWV, an acknowledged marker of large arterial stiffness [7, 34]. Even when subjects with PUA ≥ 370 µmol/l were excluded [9], PUA concentration remained a significant independent variable related with PWV. Of note, PUA levels were predominantly within the normal range in the present population: only 8/295 men and 7/311 women presented with PUA exceeding the current national upper limits of normal values. Increased arterial stiffness is a strong predictor of cardiovascular events [34], and higher uric acid concentration may thus predispose to the future development of CVD. The average PWV values in the present study were within the normal range for subjects with high-normal BP or grade 1 hypertension [35, 36].

Previous studies have linked uric acid with arterial stiffness, but the matter remains controversial [14, 37]. In a cross-sectional study comprising 651 medicated hypertensive patients and normotensive controls, serum uric acid was independently associated with carotid-femoral PWV (CF-PWV) [11]. Hypertensive patients with hyperuricemia also presented with higher CF-PWV than hypertensive patients without hyperuricemia [11]. In a large hypertensive population from China comprising 10,450 participants, of whom > 90% were receiving antihypertensive agents and ~ 50% were current smokers, serum uric acid was a risk factor for higher brachial-ankle PWV in men and women [38]. As a major difference to our study, medicated hypertensive subjects were included in the analyses of the above studies [11]. In the Framingham study, Mehta et al. found that uric acid was linked to CF-PWV in subjects with low cardiovascular risk factors, independent of BP [10]. However, when the medicated hypertensive patients were excluded from the analyses, the relation between uric acid and arterial stiffness was much weaker, albeit still statistically significant [10]. In 222 untreated hypertensive subjects without gout, PUA correlated with carotid-femoral PWV but not with 24-h systolic or diastolic BP in univariate analysis, however the association with PWV was no longer significant after correction for albuminuria and other covariates [39]. The above findings suggested that the association of uric acid with large arterial stiffness was not solely explained by the presence of hypertension. Fang et al. examined the relationship of serum uric acid with brachial-ankle PWV in apparently healthy 7025 subjects, and found a significant association in women but not in men [40]. Bian et al. found similar results in their cross-sectional analysis of 2374 subjects, in which elevated uric acid was associated with CF-PWV only in women [15]. On the other hand, in a healthy middle-aged population, serum uric acid was associated with CF-PWV in men but not in women. However, the results were not adjusted for eGFR, insulin resistance, or plasma lipids [41]. In contrast, Cicero et al. reported that uric acid was significantly associated with hypertension and carotid artery intima-media thickness, but not with CF-PWV in 619 subjects not taking antihypertensive, antidiabetic, lipid-lowering and uric-acid-lowering drugs [37].

A possible causal role for uric acid in the development of CVD is still unclear, and no consensus has been reached whether uric acid is a true risk factor or merely a marker of increased risk. However, several mechanisms have been suggested by which hyperuricemia could be linked to cardiovascular pathophysiology. Hyperuricemia may be associated with decreased nitric oxide production, and the resultant endothelial dysfunction could contribute to the development of increased arterial stiffness [16]. Experimental hyperuricemia has been characterized by decreased serum concentration of nitric oxide, while this was reversed by the lowering of uric acid levels using allopurinol [42]. Furthermore, xanthine oxidase inhibition with allopurinol was found to improve endothelium-dependent vasodilatation in the forearm of patients with heart failure and type 2 diabetes [43, 44]. However, the interpretation of the above findings is complicated by the potent antioxidant properties of uric acid both in vitro and in vivo [45, 46]. It is therefore possible that the culprit behind impaired endothelial function is the oxidative stress induced by xanthine oxidase activity and not uric acid per se. Indeed, George et al. reported that improved endothelial function induced by allopurinol was due to its ability to reduce vascular oxidative stress and not in its ability to reduce uric acid [47]. Of note, Feig et al. found that treatment of newly diagnosed hypertensive adolescents with allopurinol reduced their BP in the short term [13].

The tonometric recording of PWV is considered the gold standard method for the evaluation of large arterial stiffness [7, 34]. PWV measured by the tonometric recording of arterial pulsations was even found to predict future hypertension in elderly participants of the Framingham study [27]. Aortic-to-popliteal PWV, determined using the same impedance cardiography method that was applied in the present study, was also found to predict future hypertension in young adults [48]. Previously, we reported an excellent correlation (r = 0.82) between applanation tonometric measurements of carotid-femoral PWV and impedance cardiography measurements of aortic-to-popliteal PWV in 80 subjects [33]. The impedance-derived PWV has also been validated against the measurement of PWV using ultrasound [30]. Based on the above findings, PWV recorded using whole-body impedance cardiography can be regarded as a reliable measure of large arterial stiffness [30, 33, 48].

We found that office BP was significantly higher in Q4 versus Q1 of PUA, even after adjusting for differences in age, sex, and body mass index (Table 2). However, the present measurements performed in supine position under quiet standardized laboratory conditions did not uncover any significant differences in peripheral or central BP between the PUA quartiles. Previously, white-coat hypertension was reported to be independently associated with higher large arterial stiffness in treated hypertensive patients [49]. Furthermore, when evaluated using the ratio of pulse pressure to stroke volume, higher arterial stiffness was also significantly related to the white-coat effect in 2778 hypertensive patients [50]. As the present quartile with the highest PUA (Q4) also presented with the highest PWV, increased large arterial stiffness may be the plausible explanation for the higher office BP in these subjects versus Q1, because of the link between the white-coat effect and large arterial stiffness [49, 50].

Our study has limitations that need to be acknowledged. PUA and hemodynamics were recorded at single points of time about 12 (10, 13) days apart (mean (95% CI)), and the cross-sectional design does not allow conclusions about causality. We cannot rule out a selection bias caused by the recruitment and exclusion protocol. We applied indirect non-invasive methods requiring mathematical processing to derive PWV, stroke volume and cardiac output from the bioimpedance signal [32], and central aortic BP waveform from applanation tonometry signal [26]. Even though the methods have been validated against direct or invasive measurements [25, 29, 30], the results must be interpreted with caution. However, the approach to examine central hemodynamics, compared to just focusing on radial or brachial artery pressure, may be better related with the level of cardiovascular risk [51, 52]. Lastly, we cannot rule out potential multicollinearity problems that were not controlled for by the statistical methods, as some of the clinical characteristics and biochemical variables are strongly associated with each other.

## Conclusions

A direct association between PUA and PWV was observed in 606 normotensive and never-treated hypertensive subjects without cardiovascular disease or gout. The finding that PUA was not significantly associated with any other hemodynamic variable implies that PUA potentially plays a role in the pathogenesis of large arterial stiffness. Prospective longitudinal studies are needed to confirm the present finding.

## Data Availability

Analyses and generated datasets during the current study are not available publicly as our clinical database contains several indirect identifiers and the informed consent obtained does not allow publication of individual patient data. The datasets are available from the corresponding author on reasonable request.

## Abbreviations

BP: Blood pressure
BMI: Body mass index
CVD: Cardiovascular disease
CF-PWV: Carotid-femoral pulse wave velocity
eGFR: Estimated glomerular filtration rate
HDL: High-density lipoprotein
LDL: Low-density lipoprotein
PTH: Parathyroid hormone
PUA: Plasma uric acid
PWV: Pulse wave velocity
QUICKI: Quantitative insulin sensitivity check index
Q: Quartile
SEM: Standard error of the mean
